# Association between terbutaline sulfate plus ambroxol hydrochloride and clinical outcomes, pulmonary function, and inflammatory biomarkers in elderly patients with acute exacerbation of chronic obstructive pulmonary disease

**DOI:** 10.3389/fphar.2026.1808041

**Published:** 2026-05-20

**Authors:** Xin Yu, Haiwen Zhou, Jintao Zhu, Nini Zhang, Dexin Wang, Songbiao Zhan

**Affiliations:** Respiratory Medicine, Traditional Chinese Medical Hospital of Zhuji, Zhuji, China

**Keywords:** acute exacerbation of COPD, ambroxol hydrochloride, elderly, inflammatory markers, pulmonary function, terbutaline sulfate

## Abstract

**Background:**

Acute exacerbations of chronic obstructive pulmonary disease (AECOPD) are characterized by airflow limitation and marked airway/systemic inflammation, particularly in elderly patients.

**Objective:**

To evaluate the association between terbutaline sulfate plus ambroxol hydrochloride and clinical outcomes, pulmonary function, and inflammatory biomarkers in elderly patients with AECOPD.

**Methods:**

This single-center retrospective cohort study included hospitalized patients aged ≥60 years diagnosed with AECOPD between January 2021 and June 2023. According to the in-hospital treatment regimen recorded in the medical charts, patients were assigned to a control group receiving standard baseline treatment plus terbutaline sulfate or a combination group receiving ambroxol hydrochloride in addition to the control regimen. The primary endpoint was the change in forced expiratory volume in 1 s (FEV_1_) after 7 days of treatment. Secondary endpoints included other pulmonary function parameters, serum inflammatory and airway remodeling-related biomarkers, symptom scores, symptom relief time, length of hospital stay, adverse events, and 30-day post-discharge outcomes.

**Results:**

A total of 123 patients were included (control, n = 58; combination, n = 65). After treatment, the combination group showed greater improvement in FEV_1_ than the control group (1.28 vs. 1.12 L, P = 0.004). FEV_1_%pred, FVC, PEF, and MEF25% were also significantly better in the combination group (all P < 0.05). CRP, IL-6, IL-8, and MMP-9 decreased in both groups, with greater reductions in the combination group; EOS, TGF-β1, and bFGF decreased significantly only in the combination group (all P < 0.05). CAT and mMRC scores improved more markedly in the combination group, and symptom relief times and length of hospital stay were shorter (all P < 0.01). At 30-day follow-up, COPD-related readmission and recurrent acute exacerbation rates were lower in the combination group, whereas 30-day all-cause mortality did not differ significantly. Adverse event rates were comparable between groups. Exploratory analysis showed a higher observed overall response rate in the combination group (92.3% vs. 75.9%, P = 0.012).

**Conclusion:**

In elderly patients with AECOPD, terbutaline sulfate combined with ambroxol hydrochloride was associated with greater improvements in pulmonary function, inflammatory and airway remodeling-related biomarkers, symptom relief, and length of hospital stay than terbutaline sulfate alone.

## 1 Introduction

Chronic obstructive pulmonary disease (COPD) is a widely encountered chronic pulmonary disorder characterized by persistent respiratory complaints and sustained airflow obstruction, exhibiting a progressive and largely irreversible disease course that is commonly accompanied by airway inflammation and destruction of lung parenchymal structures (Labaki and Rosenberg, 2020). COPD poses a substantial global health challenge, causing high morbidity and mortality. Epidemiological data indicate that in 2021 there were approximately 16.9 million new cases of COPD worldwide and about 3.7 million deaths, and COPD-related mortality ranks among the highest of major chronic diseases globally (Naeem et al., 2025). With accelerated population ageing, older adults now bear the greatest burden of COPD; in China and internationally, COPD remains one of the leading causes of disability and death in the elderly (Mattiuzzi and Lippi, 2019). Patients with COPD experience a marked decline in quality of life, reduced productivity, and limited functional capacity, and a higher proportion depend on caregivers—factors that drive continuing increases in both direct and indirect management costs (Rehman et al., 2021). Consequently, COPD has become a major concern for healthcare systems and public health authorities.

Acute exacerbations of chronic obstructive pulmonary disease (AECOPD) are characterized by a sudden worsening of respiratory symptoms, commonly presenting as increased cough, greater sputum volume, or progressive dyspnea (Ritchie and Wedzicha, 2020). These episodes are frequently associated with intensification of both airway and systemic inflammatory responses, exhibit large clinical fluctuations and unpredictable recovery times, and some patients fail to return to their prior baseline within a short period (Wageck et al., 2019). Recurrent AECOPD not only accelerates the decline in pulmonary function and worsens quality of life, but also substantially increases hospitalization and mortality risk, thereby raising healthcare utilization and societal burden—effects that are particularly severe in older patients (Mantero et al., 2017). Pharmacologic management of AECOPD currently centers on bronchodilators and mucolytic agents (Agustí et al., 2023; Papadopoulou et al., 2023). Terbutaline sulfate, a selective β2-adrenergic receptor agonist, relaxes airway smooth muscle and relieves bronchospasm, and may exert modest anti-inflammatory effects; nevertheless, as monotherapy in AECOPD its ability to control symptoms and reduce airway inflammation is limited in some patients (Loureiro et al., 2021; Keränen et al., 2016). Ambroxol hydrochloride promotes sputum liquefaction and facilitates expectoration, and is a commonly used mucolytic in AECOPD treatment; however, when used alone it has certain limitations in improving pulmonary function, and some patients continue to experience dyspnea and persistent inflammation (Papadopoulou et al., 2023; Anil et al., 2024). Theoretically, the combined use of these two agents could provide complementary benefits—“bronchodilation → improved ventilation” and “mucus clearance → restored airway patency”—thereby reducing airway resistance, improving ventilation–perfusion matching, and accelerating symptom relief. Based on this pathophysiological rationale, short-term combination therapy with terbutaline sulfate and ambroxol hydrochloride may offer complementary benefits for restoring airway patency and alleviating inflammatory manifestations during acute exacerbations. Therefore, this study aimed to evaluate the association of terbutaline sulfate combined with ambroxol hydrochloride with clinical outcomes, pulmonary function, and inflammatory marker levels in elderly patients with AECOPD, thereby providing real-world observational evidence to inform treatment optimization during acute exacerbations.

## 2 Materials and methods

### 2.1 Study design and subjects

This investigation adopted a retrospective cohort design and was conducted at a single medical center. Medical records of older hospitalized patients who met the diagnostic criteria for acute exacerbations of chronic obstructive pulmonary disease were reviewed and analyzed. All eligible cases were admitted to the Department of Respiratory Medicine of the Traditional Chinese Medical Hospital of Zhuji during the period from January 2021 to June 2023. All cases meeting the inclusion criteria were grouped according to the in-hospital treatment regimen recorded in the medical charts: the control group received standard baseline treatment plus terbutaline sulfate, while the combination group received ambroxol hydrochloride in addition to the control group regimen.

Inclusion criteria: ① meeting the diagnostic criteria for AECOPD as defined in the “Guidelines for the Diagnosis and Treatment of Chronic Obstructive Pulmonary Disease (2021 revision)” issued by the Respiratory Diseases Branch of the Chinese Medical Association; ② age ≥ 60 years; ③ complete medical records and good adherence to treatment; ④ no cognitive impairment or psychiatric disorder and able to cooperate with relevant examinations and treatments (as documented in the clinical record and judged by the treating physician).

Exclusion criteria: ① coexistence of bronchial asthma, bronchiectasis, pulmonary tuberculosis, or other chronic pulmonary diseases; ② concomitant severe cardiac, hepatic, or renal dysfunction, or malignancy; ③ use of systemic glucocorticoids or immunosuppressants within the past month; ④ incomplete clinical data or insufficient follow-up information; ⑤ known allergy or intolerance to the study drug components; ⑥ presence of cognitive impairment, psychiatric disease, or poor treatment adherence.

This research protocol received formal authorization from the Ethics Committee of the Traditional Chinese Medical Hospital of Zhuji (Approval No.: 2025-025). Given that the study involved retrospective analysis of anonymized data, the Ethics Committee waived the requirement for written informed consent. All personally identifiable information was de-identified after data extraction, and access to and use of the data were strictly restricted.

### 2.2 Treatment methods

All included cases received standard background therapy according to the hospital’s routine clinical pathway for AECOPD. Background therapy included oxygen supplementation, when indicated, to correct hypoxemia; antibiotics selected and documented according to clinical indications and the hospital’s antimicrobial stewardship protocols; short-course systemic corticosteroids; expectorant therapy; and other symptomatic supportive treatments. The usual systemic corticosteroid regimen consisted of methylprednisolone 40–80 mg/day or an equivalent dose of another corticosteroid for generally 5–7 days. Antibiotics were selected based on evidence of infection, disease severity, and the hospital’s antimicrobial use protocols. Oxygen therapy was administered according to oxygen saturation, arterial blood gas analysis, and clinical manifestations. The observation period was seven consecutive days in both groups, and the same principles of background therapy were applied to both groups.

On this basis, patients in the control group received inhaled terbutaline sulfate at a dose of 0.5 mg per inhalation (one actuation), administered twice daily in accordance with the product label and routine departmental practice; additional inhalations were permitted every 4–6 h for acute symptom relief as prescribed, provided that the cumulative 24 h dose did not exceed the recommended maximum, and treatment was continued for 7 consecutive days. Patients in the combination group received the same baseline therapy and terbutaline sulfate regimen as the control group, with the addition of intravenous ambroxol hydrochloride at a dose of 30 mg diluted in 100 mL of 0.9% sodium chloride and administered by slow intravenous infusion once daily for 7 consecutive days. During the treatment period, all patients received routine health guidance, including smoking and alcohol cessation, maintenance of adequate ventilation, balanced nutrition and thermal protection. All medication administration, monitoring and follow-up data were extracted from the electronic medical records and included in the retrospective analysis.

### 2.3 Outcome measures

#### 2.3.1 Primary outcome

The primary outcome of this study was the change in forced expiratory volume in 1 s (FEV_1_) after 7 days of treatment. FEV_1_ was measured using a Jaeger MasterScreen pulmonary function system (Germany). Relevant test results before treatment and on the day continuous treatment was completed were retrospectively collected and used to compare the improvement in pulmonary function between the two groups.

#### 2.3.2 Secondary outcomes

1. Other pulmonary function parameters: These included the percent predicted forced expiratory volume in 1 s (FEV_1_%pred), forced vital capacity (FVC), FEV_1_/FVC ratio, peak expiratory flow (PEF), percent predicted peak expiratory flow (PEF%pred), and maximal expiratory flow at 25% of vital capacity (MEF25%). The results of these parameters before treatment and on the day continuous treatment was completed were retrospectively collected and used to compare changes in ventilatory function between the two groups.
2. Serum inflammatory factors and airway remodeling-related biomarkers: These included interleukin-6 (IL-6), interleukin-8 (IL-8), C-reactive protein (CRP), matrix metalloproteinase-9 (MMP-9), transforming growth factor-β1 (TGF-β1), basic fibroblast growth factor (bFGF), and peripheral blood eosinophils (EOS), and were used to assess the effects of treatment on inflammatory responses and biological processes related to airway remodeling.
3. Clinical symptom scores: These were evaluated using the Chronic Obstructive Pulmonary Disease Assessment Test (CAT) and the modified Medical Research Council dyspnea scale (mMRC). The scores recorded before treatment and on the day continuous treatment was completed were retrospectively collected. The CAT consists of eight items: (i) cough; (ii) sputum production; (iii) chest tightness; (iv) shortness of breath when walking uphill or climbing stairs; (v) limitation in household activities; (vi) confidence in leaving home; (vii) sleep quality; and (viii) energy/fatigue. Each item is scored from 0 to 5, with a total score ranging from 0 to 40; higher scores indicate more severe symptoms. The mMRC scale was used to grade dyspnea from 0 to 4, with the corresponding activity limitation recorded for each grade.
4. Time to clinical symptom recovery: The times to resolution of clinical symptoms, including high fever, chest tightness, cough, sputum production, and moist rales on lung auscultation, were collected to reflect short-term clinical recovery.
5. Length of hospital stay: The total duration of hospitalization for the current admission was collected and used to compare in-hospital outcomes between the two groups.
6. Safety outcomes: Adverse events during treatment were collected and compared, including hand tremor/trembling, palpitations, tachycardia, headache, nausea, gastrointestinal discomfort, epistaxis, rash/allergic reactions, electrolyte disturbances, and abnormalities in liver or renal function.
7. Thirty-day post-discharge follow-up outcomes: COPD-related readmission, recurrent acute exacerbation, and all-cause mortality within 30 days after discharge were collected to assess short-term prognosis.

#### 2.3.3 Exploratory outcome

The overall response rate was included as an exploratory outcome in this study to provide a supplementary reflection of overall clinical improvement after treatment. Treatment response was retrospectively assessed comprehensively according to the degree of improvement in clinical symptoms and changes in pulmonary function documented in the medical records after 7 consecutive days of treatment, and was categorized as markedly effective, effective, or ineffective. Markedly effective was defined as near-complete resolution of the main symptoms together with an increase in FEV_1_ of ≥15% from baseline or an absolute increase of ≥200 mL. Effective was defined as a marked reduction in clinical symptoms compared with baseline but not meeting the criteria for markedly effective. Ineffective was defined as improvement in symptoms and pulmonary function of <10%, or no obvious change. The overall response rate was calculated as follows: (number of markedly effective cases + number of effective cases)/total number of cases × 100%. To minimize assessment bias as much as possible, treatment response was independently evaluated by two respiratory physicians who were not involved in the patients’ clinical management, using unified prespecified criteria under de-identified conditions; any discrepancies were adjudicated by a third physician.

### 2.4 Data sources and collection

The study data were obtained from the electronic medical record system of the Traditional Chinese Medical Hospital of Zhuji (inpatient cases admitted from January 2021 to June 2023). Extracted variables included demographic information, smoking history, prior comorbidities, admission vital signs, medications and treatment course, imaging and laboratory results (including pulmonary function, inflammatory markers, and arterial blood gas), adverse events, and follow-up outcomes. Two investigators independently extracted and entered the data; disagreements were adjudicated by a third investigator. All data were de-identified and stored in a controlled database with regular backups.

### 2.5 Statistical methods

All statistical analyses were performed using SPSS version 26.0 (IBM, Armonk, NY, United States). Quantitative variables were assessed for normality and homogeneity of variance using the Shapiro–Wilk test and Levene’s test, and are presented as mean ± standard deviation (SD) or median [interquartile range, IQR], as appropriate. Categorical variables are presented as frequencies (%). Between-group comparisons of continuous variables were conducted using the independent-samples t-test or the Mann–Whitney U test, while within-group paired comparisons were performed using the paired t-test or the Wilcoxon signed-rank test. Categorical variables were compared using the χ^2^ test or Fisher’s exact test. Repeated measurements obtained at baseline and after treatment were analyzed using linear mixed-effects models to evaluate group effects, time effects, and group-by-time interaction effects; when necessary, repeated-measures analysis of variance was additionally performed with Greenhouse–Geisser correction. For comparisons involving multiple indicators within the same outcome domain, Bonferroni correction was applied to adjust for multiple comparisons and control the accumulation of type I error. Missing data were assessed before analysis; complete-case analysis was used when the proportion of missing data was <5%, whereas multiple imputation was applied when the proportion was ≥5%. In addition to reporting P values, the clinical relevance of the results was interpreted in conjunction with between-group differences, the magnitude of change, and event rates. All tests were two-sided, and a P value < 0.05 was considered statistically significant.

## 3 Results

### 3.1 Comparison of baseline clinical characteristics between the two groups

**FIGURE 1:**
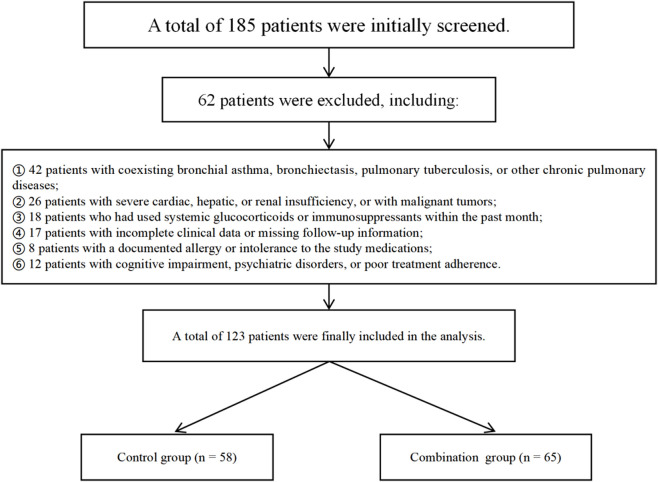
Flowchart of patient enrollment.

A total of 185 hospitalized elderly patients diagnosed with AECOPD were initially screened; after applying exclusion criteria 62 patients were excluded and 123 patients remained for analysis. According to the in-hospital treatment documented in the medical records, patients were assigned to a control group (n = 58) or a combination group (n = 65) (Figure 1). The two groups were comparable with respect to demographic and baseline clinical characteristics (Table 1). There were no significant between-group differences in age (72.1 ± 6.4 vs. 71.3 ± 7.1 years), sex distribution or body mass index (23.9 ± 3.0 vs. 24.0 ± 3.2). Disease-related baseline measures were likewise similar: median COPD duration, GOLD stage distribution, number of exacerbations and hospitalizations in the preceding year did not differ significantly between groups. Classification of smoking history, prevalence of common comorbidities (hypertension, diabetes, coronary artery disease, chronic kidney disease), and admission physiological/arterial blood gas parameters (PaO_2_, PaCO_2_), SpO_2_, body temperature and heart rate were also comparable (all P > 0.05). In summary, baseline characteristics were well balanced between the two cohorts, providing a comparable starting point for subsequent between-group comparisons of clinical outcomes and inflammatory biomarkers.

**TABLE 1:** Comparison of baseline clinical characteristics between the two groups.

Variable	Control group (n = 58)	Combination group (n = 65)	Test statistic	P value
Age (years, mean ± SD)	72.1 ± 6.4	71.3 ± 7.1	t = 0.56	0.578
Sex, n (%)				
Male	36 (62.1%)	42 (64.6%)	χ^2^ = 0.10	0.752
Female	22 (37.9%)	23 (35.4%)		
BMI (kg/m^2^, mean ± SD)	23.9 ± 3.0	24.0 ± 3.2	t = 0.28	0.782
Duration of COPD (years, median [IQR])	8.0 [5.0–12.0]	9.0 [6.0–13.0]	U = 1589.0	0.283
Pulmonary function (GOLD stage), n (%)				
I	6 (10.3%)	7 (10.8%)	χ^2^ = 0.41	0.941
II	20 (34.5%)	22 (33.8%)		
III	25 (43.1%)	30 (46.2%)		
IV	7 (12.1%)	6 (9.2%)		
Respiratory failure, n (%)	10 (17.2%)	12 (18.5%)	χ^2^ = 0.05	0.822
Number of exacerbations in the past year (median [IQR])	1 [0–2]	1 [0–2]	U = 1764.5	0.953
Hospitalizations in the past year (median [IQR])	1 [0–1]	1 [0–2]	U = 1652.0	0.603
Smoking history, n (%)				
Current smoker	18 (31.0%)	20 (30.8%)	χ^2^ = 0.06	0.972
Former smoker	28 (48.3%)	30 (46.2%)		
Never smoker	12 (20.7%)	15 (23.1%)		
Major comorbidities, n (%)				
Hypertension	30 (51.7%)	34 (52.3%)	χ^2^ = 0.01	0.934
Diabetes	12 (20.7%)	15 (23.1%)	χ^2^ = 0.09	0.762
Coronary artery disease	8 (13.8%)	10 (15.4%)	χ^2^ = 0.06	0.809
Chronic kidney disease	3 (5.2%)	4 (6.2%)	χ^2^ = 0.06	0.806
Admission SpO_2_ (%, mean ± SD)	89.6 ± 4.1	90.0 ± 4.0	t = 0.67	0.503
Admission temperature (°C, mean ± SD)	36.8 ± 0.4	36.7 ± 0.5	t = 0.85	0.398
Admission heart rate (beats/min, mean ± SD)	92.4 ± 12.1	90.2 ± 11.6	t = 0.86	0.392

### 3.2 Comparison of pulmonary function between the two groups

Before treatment, there were no statistically significant differences in pulmonary function parameters between the two groups: baseline FEV_1_ (1.00 vs. 1.02 L), FEV_1_% predicted (42% vs. 43%), FVC, and other indices were similar between groups (Table 2). After treatment, both groups exhibited significant improvement: in the control group FEV_1_ increased from 1.00 to 1.12 L (P < 0.05), while in the combination group it increased from 1.02 to 1.28 L (P < 0.05); the post-treatment FEV_1_ in the combination group was significantly higher than that in the control group (between-group P = 0.004). Similarly, control versus combination group comparisons showed greater gains in the combination group for FEV_1_% predicted (46% vs. 51%, P = 0.012), FVC (2.24 vs. 2.40 L, P = 0.037), PEF (2.92 vs. 3.12 L/s, P = 0.028), PEF% predicted (58% vs. 63%, P = 0.020), and 25% MEF (0.50 vs. 0.58 L/s, P = 0.003). Only the between-group difference in FEV_1_/FVC did not reach statistical significance (P = 0.09). These results suggest that, in this retrospective cohort, the combined regimen was associated with greater improvements across multiple ventilatory and small-airway parameters.

**TABLE 2:** Comparison of pulmonary function between the two groups.

Parameter (Unit)	Control group (n = 58)	Combination group (n = 65)	Test statistic	P value
FEV_1_ (L) (median [IQR])				
Before treatment	1.00 [0.80–1.20]	1.02 [0.84–1.24]	U = 1756	0.72
After treatment	1.12 [0.92–1.36]*	1.28 [1.05–1.52]*	U = 1460	0.004
FEV_1_%pred (%) (median [IQR])				
Before treatment	42 [36–50]	43 [37–51]	U = 1738	0.81
After treatment	46 [39–56]*	51 [42–60]*	U = 1506	0.012
FVC (L) (median [IQR])				
Before treatment	2.05 [1.80–2.45]	2.10 [1.85–2.50]	U = 1720	0.68
After treatment	2.24 [1.95–2.60]*	2.40 [2.05–2.75]*	U = 1620	0.037
FEV_1_/FVC (%) (median [IQR])				
Before treatment	43 [38–49]	44 [39–50]	U = 1742	0.59
After treatment	45 [40–51]*	47 [42–53]*	U = 1708	0.09
PEF (L/s) (median [IQR])				
Before treatment	2.60 [2.20–3.10]	2.62 [2.25–3.15]	U = 1760	0.84
After treatment	2.92 [2.50–3.40]*	3.12 [2.70–3.60]*	U = 1580	0.028
PEF%pred (%) (median [IQR])				
Before treatment	52 [44–63]	53 [45–64]	U = 1748	0.78
After treatment	58 [49–69]*	63 [54–72]*	U = 1540	0.02
MEF at 25% of FVC (L/s) (median [IQR])				
Before treatment	0.45 [0.32–0.60]	0.46 [0.33–0.62]	U = 1754	0.76
After treatment	0.50 [0.36–0.66]*	0.58 [0.40–0.74]*	U = 1402	0.003

“*” indicates a statistically significant difference compared with the pre-treatment value within the same group (P < 0.05).

### 3.3 Comparison of serum inflammatory markers and airway-remodeling–related biomarkers between the two groups

At baseline there were no statistically significant differences between the two groups in serum inflammatory markers and airway-remodeling–related biomarkers, indicating comparability at enrollment (Table 3). After treatment, CRP, IL-6, IL-8 and MMP-9 decreased significantly in both groups compared with pre-treatment values (within-group paired comparisons P < 0.05); by contrast, peripheral eosinophils (EOS), TGF-β1 and bFGF showed a significant decline only in the combination group. Between-group comparisons after treatment demonstrated that the medians of CRP, IL-6, IL-8, EOS, MMP-9, TGF-β1 and bFGF in the combination group were lower than those in the control group, and these differences were statistically significant (between-group P = 0.002, 0.005, 0.01, 0.042, <0.001, 0.045 and 0.011, respectively) (see Table 3). In summary, combination therapy was associated with greater reductions in markers of inflammation and airway remodeling than monotherapy in this retrospective analysis, and most indices reached statistical significance in between-group comparisons.

**TABLE 3:** Comparison of serum inflammatory factors and airway remodeling-related markers between the two groups.

Indicator (Unit)	Control group (n = 58)	Combination group (n = 65)	Test statistic	P value
CRP (mg/L, median [IQR])				
Before treatment	45.0 [22–88]	42.0 [20–80]	U = 1790	0.7
After treatment	18.0 [8–38]*	10.0 [4–22]*	U = 1305	0.002
IL-6 (pg/mL, median [IQR])				
Before treatment	25.0 [10–60]	22.0 [9–55]	U = 1735	0.65
After treatment	11.0 [5–24]*	7.5 [3–15]*	U = 1360	0.005
IL-8 (pg/mL, median [IQR])				
Before treatment	110 [45–250]	120 [50–280]	U = 1768	0.68
After treatment	50 [20–120]*	30 [12–80]*	U = 1408	0.01
EOS (10^9/L^, median [IQR])				
Before treatment	0.16 [0.08–0.32]	0.15 [0.07–0.30]	U = 1802	0.81
After treatment	0.14 [0.07–0.28]	0.11 [0.05–0.20]*	U = 1568	0.042
MMP-9 (ng/mL, median [IQR])				
Before treatment	80 [40–160]	85 [42–170]	U = 1750	0.74
After treatment	60 [30–120]*	40 [20–90]*	U = 1285	<0.001
TGF-β1 (pg/mL, median [IQR])				
Before treatment	400 [280–620]	420 [300–640]	U = 1740	0.72
After treatment	360 [250–540]	340 [220–500]*	U = 1600	0.045
bFGF (pg/mL, median [IQR])				
Before treatment	45 [18–120]	50 [20–140]	U = 1756	0.73
After treatment	30 [12–80]	18 [8–55]*	U = 1415	0.011

“*” indicates a statistically significant difference compared with the pre-treatment value within the same group (P < 0.05).

### 3.4 Comparison of clinical symptom scores, time to symptom resolution, and therapeutic response between the two groups

This retrospective analysis showed no significant differences between the two groups in baseline CAT total scores or mMRC grade distribution (Table 4). After treatment, CAT total scores decreased in both groups (both within-group P < 0.05), but the reduction was greater in the combination group (median: 14 (Anil et al., 2024; Loureiro et al., 2021; Keränen et al., 2016; Spannella et al., 2019; van Dam Van Isselt et al., 2019; Elkomy et al., 2022; Kantar et al., 2025; Zhang et al., 2016; Machida et al., 2020) in the control group vs. 10 (Mantero et al., 2017; Agustí et al., 2023; Papadopoulou et al., 2023; Anil et al., 2024; Loureiro et al., 2021; Keränen et al., 2016; Spannella et al., 2019; van Dam Van Isselt et al., 2019) in the combination group; U = 1286, P = 0.001). The distribution of mMRC grades also shifted toward milder dyspnea, and after treatment, the proportions of patients who were asymptomatic (grade 0) or had mild dyspnea (grade 1) were significantly higher in the combination group than in the control group (P < 0.05).

**TABLE 4:** Comparison of clinical symptom scores, symptom resolution time, and therapeutic outcomes between the two groups.

Variable	Control group (n = 58)	Combination group (n = 65)	Test statistic	P value
CAT total score (0–40), median [IQR]				
Before treatment	18 [15–22]	19 [16–24]	U = 1720	0.48
After treatment	14 [10–18]*	10 [7–14]*	U = 1286	0.001
mMRC grade (0–4), n (%) before treatment				
Grade 0	2 (3.4%)	3 (4.6%)	χ^2^ = 0.23	0.89
Grade 1	10 (17.2%)	12 (18.5%)		
Grade 2	25 (43.1%)	30 (46.2%)		
Grade 3	16 (27.6%)	15 (23.1%)		
Grade 4	5 (8.6%)	5 (7.7%)		
After treatment				
Grade 0	8 (13.8%)	22 (33.8%)*	χ^2^ = 8.50	0.014
Grade 1	20 (34.5%)	28 (43.1%)*		
Grade 2	18 (31.0%)	10 (15.4%)		
Grade 3	8 (13.8%)	3 (4.6%)		
Grade 4	4 (6.9%)	2 (3.1%)		
Time to resolution of primary symptoms (days, median [IQR])				
Cough resolution time	5 [3–8]	3 [2–5]	U = 1410	0.002
Sputum resolution time	6 [4–9]	3 [2–6]	U = 1378	0.001
Relief time of dyspnea/shortness of breath	4 [2–6]	2 [1–4]	U = 1360	0.001
Disappearance of pulmonary moist rales	3 [2–6]	2 [1–4]	U = 1490	0.01
Resolution of high fever	2 [1–3]	1 [1–2]	U = 1620	0.03
Length of hospital stay (days, median [IQR])	9 [7–12]	7 [6–10]	U = 1402	0.005
Therapeutic response, n (%)				
Markedly effective	8 (13.8%)	20 (30.8%)	χ^2^ = 8.50	0.014
Effective	36 (62.1%)	40 (61.5%)		
Ineffective	14 (24.1%)	5 (7.7%)		
Overall response rate, n (%)	44 (75.9%)	60 (92.3%)	χ^2^ = 6.34	0.012

“*” indicates a statistically significant difference compared with pre-treatment values within the same group (P < 0.05).

Regarding time to clinical symptom relief, the median time to symptom resolution was shorter in the combination group than in the control group. Specifically, the time to resolution of cough was 3 (Naeem et al., 2025; Mattiuzzi and Lippi, 2019; Rehman et al., 2021; Ritchie and Wedzicha, 2020) days versus 5 (Mattiuzzi and Lippi, 2019; Rehman et al., 2021; Ritchie and Wedzicha, 2020; Wageck et al., 2019; Mantero et al., 2017; Agustí et al., 2023) days (U = 1410, P = 0.002), the time to resolution of sputum production was 3 (Naeem et al., 2025; Mattiuzzi and Lippi, 2019; Rehman et al., 2021; Ritchie and Wedzicha, 2020; Wageck et al., 2019) days versus 6 (Rehman et al., 2021; Ritchie and Wedzicha, 2020; Wageck et al., 2019; Mantero et al., 2017; Agustí et al., 2023; Papadopoulou et al., 2023) days (U = 1378, P = 0.001), and the time to resolution of dyspnea was 2 (Labaki and Rosenberg, 2020; Naeem et al., 2025; Mattiuzzi and Lippi, 2019; Rehman et al., 2021) days versus 4 (Naeem et al., 2025; Mattiuzzi and Lippi, 2019; Rehman et al., 2021; Ritchie and Wedzicha, 2020; Wageck et al., 2019) days (U = 1360, P = 0.001). In addition, the times to resolution of moist rales and fever were also shorter in the combination group than in the control group (U = 1490, P = 0.010; U = 1620, P = 0.030, respectively). The length of hospital stay was likewise shorter in the combination group, with a median duration of 7 (Wageck et al., 2019; Mantero et al., 2017; Agustí et al., 2023; Papadopoulou et al., 2023; Anil et al., 2024) days compared with 9 (Mantero et al., 2017; Agustí et al., 2023; Papadopoulou et al., 2023; Anil et al., 2024; Loureiro et al., 2021; Keränen et al., 2016) days in the control group (U = 1402, P = 0.005). As an exploratory finding, both the number of markedly improved cases and the overall response rate were higher in the combination group than in the control group (marked improvement rate: 30.8% vs. 13.8%, χ^2^ = 8.50, P = 0.014; overall response rate: 92.3% vs. 75.9%, χ^2^ = 6.34, P = 0.012), as shown in Table 4.

### 3.5 Comparison of 30-day follow-up outcomes between the two groups

A total of 115 patients completed the 30-day follow-up, including 54 in the control group and 61 in the combination group. Analysis of patients who completed the 30-day follow-up showed that both the COPD-related readmission rate and the recurrent acute exacerbation rate were lower in the combination group than in the control group. The 30-day COPD-related readmission rate was 4.9% (3/61) in the combination group, significantly lower than 18.5% (10/54) in the control group (P = 0.036). The 30-day recurrent acute exacerbation rate was also lower in the combination group than in the control group [8.2% (5/61) vs. 22.2% (12/54), P = 0.039]. No statistically significant difference was observed between the two groups in 30-day all-cause mortality [0.0% (0/61) vs. 1.9% (1/54), P = 0.470]. These short-term between-group differences should be interpreted cautiously because the analysis was limited to patients who completed follow-up and did not adjust for post-discharge management or other potential confounders, as shown in Table 5.

**TABLE 5:** Comparison of 30-day follow-up outcomes between the two groups.

Outcome	Control group (n = 54)	Combination group (n = 61)	Test statistic	P value
30-day COPD-related readmission rate	10/54 (18.5%)	3/61 (4.9%)	—	0.036
30-day recurrent acute exacerbation rate	12/54 (22.2%)	5/61 (8.2%)	—	0.039
30-day all-cause mortality rate	1/54 (1.9%)	0/61 (0.0%)	—	0.47

A total of 115 patients completed the 30-day follow-up, including 54 in the control group and 61 in the combination group. COPD-related readmission was defined as rehospitalization due to AECOPD, or worsening of related respiratory symptoms. Recurrent acute exacerbation was defined as symptom worsening requiring additional antibiotics and/or systemic corticosteroids, emergency treatment, or hospitalization. “—” indicates that Fisher’s exact test was used.

### 3.6 Comparison of adverse event occurrence between the two groups

There was no statistically significant difference in the total occurrence of adverse events between the two treatment cohorts (P > 0.05; refer to Table 6). In the control group, 13 patients experienced ≥1 adverse event, with an overall incidence of 22.4%; in the combination group there were 19 cases, an incidence of 29.2%, and the difference between groups was not statistically significant (χ^2^ = 0.78, P = 0.38). By specific categories, palpitations occurred in 6 patients (10.3%) in the control group and 9 patients (13.8%) in the combination group (χ^2^ = 0.36, P = 0.55); hand tremor/shaking occurred in 5 patients (8.6%) and 8 patients (12.3%), respectively (χ^2^ = 0.43, P = 0.51). The frequencies of headache and nausea/vomiting were both low (headache: 6.9% vs. 9.2%, P = 0.63; nausea/vomiting: 5.2% vs. 7.7%, P = 0.56). Electrolyte disturbances, hepatic and renal function abnormalities, allergic reactions, and epistaxis were all uncommon events, and none of these individual categories showed significant differences between the two groups (all P > 0.05). Although the combination therapy group exhibited a marginally greater number of adverse events compared with the control group, neither the incidence of individual events nor the overall frequency demonstrated statistical significance.

**TABLE 6:** Comparison of adverse events between the two groups.

Adverse event category	Control group n (%)	Combination group n (%)	Test statistic	P-value
Palpitations	6 (10.3%)	9 (13.8%)	χ^2^ = 0.36	0.55
Tremor	5 (8.6%)	8 (12.3%)	χ^2^ = 0.43	0.51
Headache	4 (6.9%)	6 (9.2%)	χ^2^ = 0.24	0.63
Nausea/Vomiting	3 (5.2%)	5 (7.7%)	χ^2^ = 0.34	0.56
Hypokalemia	2 (3.4%)	4 (6.2%)	—	0.68
Abnormal liver function	2 (3.4%)	3 (4.6%)	—	0.71
Abnormal renal function	1 (1.7%)	2 (3.1%)	—	0.62
Allergic reaction	1 (1.7%)	2 (3.1%)	—	0.62
Epistaxis	2 (3.4%)	3 (4.6%)	—	0.71
Overall incidence of adverse events	13 (22.4%)	19 (29.2%)	χ^2^ = 0.78	0.38

The overall incidence of adverse events refers to the proportion of patients in each group who experienced at least one of the listed adverse events.“—” indicates that Fisher’s exact test was used.

## 4 Discussion

Management of acute exacerbations of chronic obstructive pulmonary disease (COPD) in elderly patients poses substantial challenges, primarily due to reduced physiological reserve and multiple comorbidities in this population; these patients face higher mortality risk and greater care needs during exacerbations, and their clinical and functional status are often impaired, necessitating optimized comprehensive treatment strategies (Spannella et al., 2019; Van Dam Van Isselt et al., 2019). Terbutaline sulfate, a selective β2-adrenoceptor agonist, has been shown to produce bronchodilation and effectively relieve bronchospasm and respiratory distress; its safety and efficacy in prehospital and in-hospital emergency management have been substantiated to a certain extent (Elkomy et al., 2022). Ambroxol hydrochloride, as a mucolytic agent, is widely used in chronic airway diseases; by promoting mucus clearance and exerting anti-inflammatory and antioxidant effects, it can improve sputum characteristics, enhance ciliary clearance, and mitigate airway inflammatory responses, and is considered an important adjunctive therapy in elderly AECOPD (Kantar et al., 2025; Zhang et al., 2016). This study systematically evaluated the associations of terbutaline sulfate combined with ambroxol hydrochloride with clinical outcomes and biomarkers in elderly patients with acute exacerbations of COPD. The results showed that, compared with terbutaline sulfate alone, the combination regimen was associated with greater improvements in pulmonary function recovery, reductions in inflammatory marker levels, and symptom relief, while the incidence of adverse events was comparable between the two groups. However, given the retrospective and non-randomized design, these findings should be interpreted as observational associations rather than evidence of definitive superiority or causal treatment effects. Furthermore, from the perspectives of functional improvement and inflammation control, this study provides additional real-world observational data supporting further evaluation of combination pharmacotherapy for acute exacerbations of COPD, thereby expanding potential clinical intervention options and informing treatment optimization.

In acute exacerbations of COPD, pulmonary function parameters such as FEV_1_, FVC and PEF carry important value for clinical decision-making; these indices not only reflect the severity of airflow limitation but also constitute key criteria for disease staging and prognostic assessment (Machida et al., 2020; Halpin et al., 2017). The present study showed that the combination of terbutaline sulfate and ambroxol hydrochloride was associated with more pronounced improvements in multiple pulmonary function parameters, including FEV_1_, FVC, and PEF, than the regimen with terbutaline sulfate alone, suggesting that the synergistic effects of bronchodilation and mucus clearance may be associated with improved airway patency during the acute phase. Terbutaline sulfate, a selective β_2_-adrenergic receptor agonist, promotes relaxation of airway smooth muscle and rapidly relieves airflow obstruction; it also possesses anti-inflammatory potential by modulating the cAMP signaling pathway to suppress the production of inflammatory mediators (Beng et al., 2019; Keränen et al., 2017). Ambroxol hydrochloride reduces sputum viscosity and attenuates airway inflammation, markedly enhancing mucociliary clearance and expectoration, with broad effects on mucus liquefaction and inflammatory responses; their combined use thus exhibits multi-mechanistic synergistic advantages (Zhang et al., 2016). At the level of small-airway function in COPD, our combined regimen showed a degree of novelty in improving small-airway–related indices such as PEF and MEF25%, complementing (Alobaidi et al., 2020) exploration of the diagnostic and therapeutic value of small-airway physiological assessment in the acute phase of AECOPD, and indicating that small-airway measurements may serve as sensitive markers of treatment effect. Therefore, terbutaline sulfate combined with ambroxol hydrochloride may be associated with improved recovery of pulmonary function and reduced disease burden in elderly patients with COPD during acute exacerbations, but these observations should not be interpreted as proof of a causal treatment benefit.

Inflammatory cytokines and markers of airway remodeling play a pivotal role in acute exacerbations of COPD and in disease progression. Studies have shown that inflammatory mediators such as CRP, IL-6 and IL-8 can drive chronic airway inflammation, thereby affecting airflow limitation and promoting sustained disease progression (Fermont et al., 2019; Brightling and Greening, 2019). At the same time, airway-remodeling–related molecules such as MMP-9, TGF-β1 and bFGF are key mediators of structural airway changes and fibrosis; upregulation of TGF-β1 and MMP-9 is closely associated with increased airway wall thickness and greater severity of obstruction, indicating that these factors participate in COPD-related airway remodeling and functional impairment (Górka et al., 2016; Wu et al., 2023). On this basis, the present study found that the combination therapy group showed significantly greater reductions in inflammatory factors and airway remodeling-related markers than the control group, with the most prominent changes observed in EOS, TGF-β1, and bFGF, suggesting that terbutaline sulfate combined with ambroxol hydrochloride was associated with more marked decreases in inflammatory and airway remodeling-related biomarkers. Consistent with previous studies, Xu and Pang (2023) reported that combined LABA and LAMA therapy can significantly reduce levels of inflammatory mediators such as IL-6 and IL-8 and improve lung function, with combination therapy outperforming monotherapy, while Lin et al. (2017) showed that ambroxol hydrochloride combined with lung-protective therapy can effectively downregulate TNF-α and IL-1β, attenuate the inflammatory response and enhance pulmonary function. In elderly patients with acute exacerbations of COPD, the present study found that combination therapy with terbutaline sulfate and ambroxol hydrochloride was associated with more pronounced reductions in inflammation-related markers. At the molecular level, terbutaline sulfate, as a β2-adrenergic receptor agonist, can inhibit ERK signaling in macrophages via a cAMP-dependent pathway, downregulate proinflammatory gene expression such as TNF-α and MCP-1, and thereby reduce inflammatory cell recruitment (Keränen et al., 2017); ambroxol hydrochloride, by promoting mucus clearance and ciliary motility, indirectly suppresses release of inflammatory mediators and inhibits expression of remodeling-related molecules (Zhang et al., 2016). Therefore, combination therapy appears to be associated with certain advantages in controlling acute-phase inflammatory responses and improving airway remodeling-related indicators, although causal inference cannot be established from the present study.

Elderly patients with acute exacerbations of COPD commonly present with symptoms such as cough, sputum production, dyspnea, high fever, and moist rales on lung auscultation. These manifestations not only directly impair daily functioning and quality of life, but are also closely associated with prolonged hospitalization and delayed recovery. The speed of symptom relief is therefore considered a key factor influencing prognosis (Lee et al., 2017; Kokturk et al., 2019). The present study showed that, compared with the control group, patients treated with terbutaline sulfate combined with ambroxol hydrochloride had significantly shorter times to resolution of clinical symptoms such as cough, sputum production, and dyspnea, greater improvements in CAT and mMRC scores, and a shorter length of hospital stay. As a supplementary exploratory finding, the combination group also had a higher number of markedly improved cases and a higher overall response rate than the control group, suggesting that the combination regimen was associated with faster symptom relief and overall recovery in this cohort. Similarly, Beier et al. (2017) reported that combined bronchodilator therapy was associated with improved morning and nighttime symptoms, shorter symptom duration, and better recovery of activity capacity in patients with COPD, with efficacy outcomes reported to be more favorable than those of monotherapy and a comparable incidence of adverse events. These therapeutic benefits may stem from terbutaline sulfate’s bronchodilatory action on airway smooth muscle, effectively reducing airway resistance (Elkomy et al., 2022), while ambroxol hydrochloride contributes by modulating mucus properties, enhancing ciliary motility, and suppressing inflammatory responses, thereby synergistically improving the airway milieu (Wu et al., 2025). The combination of these two agents may therefore contribute to improved airway patency through multiple mechanisms and may be associated with faster symptom relief. In addition, the 30-day follow-up results of this study showed lower rates of COPD-related readmission and recurrent acute exacerbation in the combination group, which may indicate a potentially more favorable short-term clinical course; however, these findings should not be interpreted as evidence of a causal prognostic benefit.

Therefore, early symptom control may be associated with a lower risk of recurrent acute exacerbations and improved subsequent disease management and quality of life, but this hypothesis requires confirmation in prospective studies.

Among individuals with acute COPD exacerbations receiving concurrent bronchodilator and mucolytic therapy, observed side effects primarily consisted of palpitations, tremors, and gastrointestinal symptoms; these reactions are of particular importance when evaluating clinical safety in elderly patients (Alobaidi et al., 2020; Tenório et al., 2021). The present investigation indicated that, while the combination therapy group experienced a marginally greater occurrence of adverse reactions compared with the control group, this discrepancy was not statistically significant; individual symptoms such as palpitations and tremor also did not show a marked increase, suggesting an overall favorable safety profile. Compared with the systematic review by Gong et al. (2023) on the safety of fixed-dose LABA/LAMA combinations, which reported generally good tolerability for multiple classes of bronchodilator combinations and no significant rise in serious adverse events or cardiovascular events—findings consistent with those of the present study—our results align with existing evidence. The safety analysis by Rogliani et al. (2018) concerning LABA/LAMA versus LABA/ICS regimens further indicates that combination strategies did not produce a significant elevation in adverse-event risk, supporting their suitability for older patients and those with multiple comorbidities. Therefore, within the framework of safety evaluation, no apparent increase in the risk of adverse events was observed with terbutaline sulfate combined with ambroxol hydrochloride in elderly patients with acute exacerbations of COPD. However, given that the study population consisted of elderly patients, even mild adverse events may affect overall treatment tolerability and clinical management. In addition, due to the retrospective study design, the causal attribution of some adverse events to the study medications could not be fully established.

This study has several limitations. First, this was a single-center retrospective cohort study that included 123 patients, with a relatively small sample size, and all cases were derived from the same regional medical center, which may limit the external generalizability of the findings. Second, the sample size was determined by hospitalized cases that met the inclusion and exclusion criteria and had complete records during the study period, and no *a priori* sample size calculation was performed; therefore, the statistical power and robustness of the conclusions may have been affected. Third, retrospective data extraction inevitably introduced risks of selection bias and information bias, and some data on treatment adherence, adverse events, and follow-up outcomes relied primarily on medical records, which may have influenced data completeness and accuracy. In addition, treatment allocation was not randomized, and clinicians may have made individualized medication decisions according to disease severity and actual clinical needs; therefore, residual confounding cannot be fully excluded. Although background treatment was described and generally followed the same clinical pathway, variability in systemic corticosteroid dosing, possible differences in corticosteroid type and duration, and heterogeneity in antibiotic selection based on infection-related features and illness severity may still have acted as important confounders. Moreover, no additional methods, such as propensity score matching or inverse probability weighting, were used to further adjust for baseline differences and selection bias between groups. Given the limited sample size, more complex multivariable outcome models were not constructed in order to avoid model instability and overfitting due to insufficient event numbers. Accordingly, all between-group differences observed in this study should be interpreted as associations rather than direct evidence of causal treatment effects.

This study also included the overall response rate as an exploratory outcome; however, this endpoint was based on a composite assessment of symptom improvement and changes in pulmonary function, involved a certain degree of subjectivity, and has not been widely validated as a standard endpoint in AECOPD research. Although 30-day post-discharge follow-up outcomes were further analyzed, the follow-up duration remained relatively short and was insufficient to comprehensively evaluate the longer-term clinical course of the combination regimen. In addition, outcomes such as 30-day readmission and recurrent acute exacerbation may also have been influenced by factors that were not adjusted for in the present study, including comorbidity burden, post-discharge management, maintenance inhaled therapy, medication adherence, rehabilitation, and follow-up intensity. Therefore, the observed differences in 30-day outcomes should be interpreted cautiously and should not be regarded as definitive evidence of a prognostic benefit of the combination regimen. Future multicenter, large-sample, prospective studies with longer follow-up and more rigorous control of in-hospital and post-discharge confounding factors are warranted to further evaluate the clinical value and safety of this combination regimen in elderly patients with AECOPD.

## 5 Conclusion

In elderly patients with AECOPD, terbutaline sulfate combined with ambroxol hydrochloride, compared with terbutaline sulfate alone, was associated with greater improvements in pulmonary function, greater reductions in inflammatory and airway remodeling-related biomarkers, faster symptom relief, and a shorter length of hospital stay. The regimen was generally well tolerated and was also associated with lower rates of 30-day COPD-related readmission and recurrent acute exacerbation. Given the retrospective, non-randomized design, these findings should be interpreted cautiously as observational associations rather than definitive evidence of superiority or causal efficacy. These findings warrant confirmation in larger cohorts and prospective studies.

## Data Availability

The raw data supporting the conclusions of this article will be made available by the authors, without undue reservation.
